# *Clostridium perfringens*—Opportunistic Foodborne Pathogen, Its Diversity and Epidemiological Significance

**DOI:** 10.3390/pathogens12060768

**Published:** 2023-05-26

**Authors:** Tomasz Grenda, Aleksandra Jarosz, Magdalena Sapała, Anna Grenda, Ewelina Patyra, Krzysztof Kwiatek

**Affiliations:** 1 Department of Hygiene of Animal Feeding Stuffs, National Veterinary Research Institute in Pulawy, Partyzantow 57, 24-100 Pulawy, Poland; magdalena.sapala@piwet.pulawy.pl (M.S.); ewelina.patyra@piwet.pulawy.pl (E.P.); kwiatekk@piwet.pulawy.pl (K.K.); 2 Department of Pneumonology, Oncology and Allergology, Medical University in Lublin, Jaczewskiego 8, 20-950 Lublin, Poland; anna.grenda@umlub.pl

**Keywords:** anaerobe, *Clostridium perfringens*, *Clostridium*, epidemiology, infection, toxinotype, toxins, virulence

## Abstract

The *C. perfringens* species is associated with various environments, such as soils, sewage, and food. However, it is also a component of the gastrointestinal (GI) microflora (i.e., microbiota) of sick and healthy humans and animals. *C. perfringens* is linked with different systemic and enteric diseases in livestock and humans, such as gas gangrene, food poisoning, non-foodborne diarrhoea, and enterocolitis. The strains of this opportunistic pathogen are known to secrete over 20 identified toxins that are considered its principal virulence factors. *C. perfringens* belongs to the anaerobic bacteria community but can also survive in the presence of oxygen. The short time between generations, the multi-production capability of toxins and heat-resistant spores, the location of many virulence genes on mobile genetic elements, and the inhabitance of this opportunistic pathogen in different ecological niches make *C. perfringens* a very important microorganism for public health protection. The epidemiological evidence for the association of these strains with *C. perfringens*–meditated food poisoning and some cases of non-foodborne diseases is very clear and well-documented. However, the genetic diversity and physiology of *C. perfringens* should still be studied in order to confirm the importance of suspected novel virulence traits. A very significant problem is the growing antibiotic resistance of *C. perfringens* strains. The aim of this review is to show the current basic information about the toxins, epidemiology, and genetic and molecular diversity of this opportunistic pathogen.

## 1. Introduction

In October 1891, the American pathologist, physician, and bacteriologist William H. Welch performed an autopsy on a 38-year-old man. The autopsy report showed that the body was still warm despite the weather being cool. The patient was diagnosed with chronic pulmonary tuberculosis, miliary tuberculosis, and a large saccular aneurysm of the ascending aortic arch that had ruptured in two places through the anterior chest wall. Welch observed that the body showed extensive emphysematous crackles that were diffuse and symmetrical. Gas leakage was also observed at the circular skin openings over a tumour on the anterior right side of the chest. Welch observed gas bubbles in dissected veins and arteries without any incisions in them. Bubbles of gas were also observed in the spleen, heart, kidneys, and liver. Doctor Welch revealed bacilli after a microscopic evaluation of the blood and organs. He described the bacilli as about 3 to 5 µm in length, slightly round with some square ends, and in pairs but not long chains. Capsules were also noticed. No other bacterial species were observed in slide preparations made from the tissues of the patient. The discovered bacilli were not able to move, and they possessed spores. It was possible to stain them with aniline dyes [1].

Based on the above-mentioned observations by Welch in 1891 [1], a full description of this case report, and subsequent research by Welch and Nuttall in 1892 [1,2], a new microorganism was described and named *Bacillus aerogenes capsulatus*, but the capsule was not always present in the cells. In addition, it was called *Bacillus welchii*. Dr Welch had also taken into consideration *Bacillus pneumathaemia*, *Bacillus sanguinis aerogenes*, *Bacillus aerogenes*, *Bacillus aerogenes cadaveris*, and *Bacillus pneumathaemia*. The researcher also conducted experiments on rabbits with cultures of the discovered microorganism and observed that when pathogenic cells had spread through the circulatory system, it took much longer for gas to spread through the vessels in deceased animals and consequently into the organs. Furthermore, the conducted experiments demonstrated that the new bacteria could grow in fluid cultures before the oxygen was completely absorbed (the experiments were conducted using Buchner’s method). Welch hypothesised that the presence of thrombi, dead tissues, or cavities could cause the oxygen concentration to decrease, and in such protected conditions, the bacilli were able to grow and produce gas [2,3]. Dr Welch’s experiments were noted in the United States Surgeon General’s report on the US Army’s medical preparedness and reaction to medical problems with injuries during World War I. The occurrence of *Bacillus welchii* came to be associated with gas gangrene cases caused by chemical or mechanical injuries on the battlefields [1,4].

As mentioned above, Welch and Nuttal [1,2] named the discovered microorganism *Bacillus aerogenes capsulatus* nov. spec. Fränkel suggested another name: *Bacillus phlegmonis emphysematosae* [5]. It was further named *Bacillus perfringens*, from the Latin name *perfringere*, which means ‘to break’, because of its ability to produce abundant quantities of gas, which could disrupt agar media surfaces. After that, the name *Bacillus welchii* was used, coming from the surname of Dr Welch. The name *Clostridium* (from the Greek name kloster, Latinised into *Clostridium,* which means spindle-shaped) formally came to be used in 1920, and both *Clostridium perfringens* and *Clostridium welchii* were reported by the Society for American Bacteriologists in its bacterial classification [6]. Currently, this microorganism is known as *Clostridium perfringens* [5].

## 2. *Clostridium perfringens* Organism and Its Toxins

The *C. perfringens* species is linked with various environments, such as soils, food, and sewage. However, it is also a component of the gastrointestinal (GI) microflora (i.e., microbiota) of sick and healthy humans and animals. Next-generation sequencing (NGS) technology revealed evidence of *C. perfringens* in the mummified gastrointestinal tract of the more than 5000-year-old ‘Tyrolean Iceman’, commonly known as Ötzi, found in an alpine glacier in 1991 [7]. *C. perfringens* is associated with various serious systemic and intestinal diseases of humans and animals, such as gas gangrene, food poisoning, diarrhoea not related to food, and enteritis. The strains of this opportunistic pathogen are known to secrete more than 20 identified toxins, which are considered the main virulence factors of disease course.

The strains of *C. perfringens* belong to the anaerobic bacteria community. However, they can still survive in the presence of oxygen and under low concentrations of superoxide [8,9]. It has been noticed that *C. perfringens* can potentially survive in aerobic environments (such as surfaces in hospital wards—they are not sensitive to oxygen) and can initiate disease course in aerophilic environments (i.e., adult/pre-term infant intestines) and in oxygen-exposed tissues (gas gangrene), which may facilitate bacterial host-to-host transmission [9].

The toxinotyping of *C. perfringens* relies on it carrying the *cpa/plc*, *cpb*, *etx*, *iap/ibp/itx*, *cpe*, and *netB* genes, which encode the six toxins α, β, ε, ι, CPE, and NetB, respectively. In the 1930s and 1940s, the scheme of toxinotyping was developed based on Wilsdon’s system with further modifications. In this scheme, five toxinotypes were recognised, which are able to produce a combination of the major toxins, α-, ι-, β-, and ε-toxins [10]. In 2018, Rood et al. [5] proposed a new classification extending the existing scheme to seven toxinotypes (A–G, Table 1). Table 1 also indicates the most frequent diseases associated with each type. The strains with the ability to produce α and CPE toxins were classified as type F, and strains able to produce α- and NetB toxins are considered type G.

**Figure 1:**
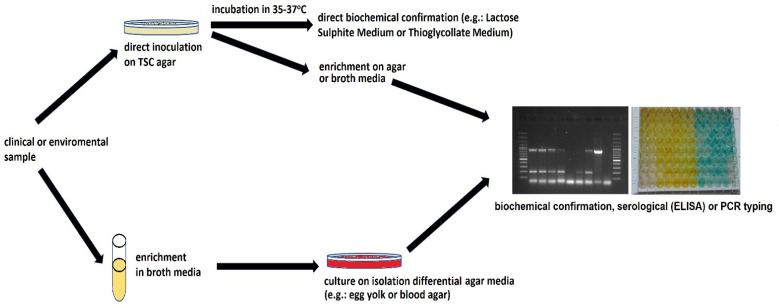
Scheme of *C. perfringens* detection.

Most often, *Clostridium perfringens* is detected using classic culture methods, and isolates are assessed in terms of features typical of the species (Figure 1). *C. perfringens* produces oval spores that are extremely rarely observed in vitro [13] and can usually be inactivated after 2–3 min of heating at 100 °C. However, spores of some strains can survive this treatment for up to 5 h [14,15]. This high thermal resistance can be associated with epidemic strains with the highest lethal potency. Li et al. [16,17] noticed that *C. perfringens* spores of the Ssp4 (small acid soluble protein 4) variant are extremely heat-resistant. *C. perfringens* is able to multiply over a wide range of temperatures, from 20 to even 50 °C [18]. This microorganism has a short generation time (8–10 min) and relatively high oxygen tolerance [8]. The optimal pH for growth and toxin production is 6.5 to 7.5 [19]. According to the AOAC, the detection and enumeration of *C. perfringens* are recommended to be conducted on TSC (tryptose sulfite cycloserine agar) medium [20]. The recommended temperature is 35–37 °C. Typically, *C. perfringens* colonies on TSC with egg yolk are black, with a 2–4 mm opaque zone formed as a result of lecithinolysis. Confirmation of belonging to *C. perfringens* species is conducted by Gram staining and testing on a lactose-gelatin medium by checking its gas production and lactose fermentation ability and movement capacity. Usually, *C. perfringens* strains are considered immobile. However, Varga et al. [21] stated that they produce type IV pili (TFP) and move with an unusual form of gliding motility, involving groups of densely packed cells moving away from the edge of the colony in curvilinear flares. The cultured isolates formed regular, round colonies with precipitation zones, indicating lecitinolytic properties.

Currently, the culture and biochemical result obtained using the culture method is usually verified with a PCR test [13,22]. The detection scheme of *C. perfringens* is presented in Figure 1. The identification is confirmed by the detection of the sequences of genes encoding the production of the main toxins, which make it possible to classify suspected strains to known toxinotypes: *plc* or *cpa* (α-toxin); *cpb* (β-toxins); *etx* (ε-toxins); *iap, ibp, itx* (ι-toxins); *cpe* CPE and *netB* (NetB). Furthermore, specific primers are used to detect genes encoding the minor toxins or conservative 16S rRNA sequences [23].

In addition, *C. perfringens* toxins can be detected using immunological methods, such as ELISA detection tests for, e.g., enterotoxin during poisoning of the digestive tract [24], latex agglutination [25], or reverse passive latex agglutination [26]. Determining the enterotoxicity of faecal isolates or food requires the induction of strain sporulation in special substrates that stimulate sporulation and the production of toxins such as Ellner’s broth, AEA, or Duncan-Strong medium [27,28].

Twenty toxins of *C. perfringens* have been described. However, the most important from the epidemiological and pathomorphological points of view are the major toxins and Perfringolysin O [29], described below and listed in Table 2:

- CPA (α-toxin) has enzyme activity able to degrade cell membranes by breaking down phosphatidylcholine and sphingomyelin and inhibiting the maturation of neutrophils as well as activating arachidonic acid metabolism. This then leads to vasoconstriction and platelet aggregation [30]. Consequently, the CPA toxin causes impairment of the innate immune response [31,32]. It is an extremely important factor in gas gangrene [5].
- CPB (β-toxin) is known as a pore-forming toxin able to bind endothelial cells and cause neurotoxic symptoms by releasing substance P [31]. Furthermore, it is considered to play a critical role in necrotising enterocolitis [33].
- ETX (ε-toxin) is the main aetiological factor of haemorrhagic enteritis and enterotoxaemia in sheep [34]. It becomes active after the action of enteric proteases and causes an increase in intestinal permeability. It is a cause of perivascular oedema with rapid cellular oedema. Moreover, it is able to accumulate in the kidneys and brain. The mechanism of the mentioned activity has not been elucidated until now [35,36].
- ITX (ι-toxin) is known as a binary toxin that is able to produce two distinct proteins: Ia and Ib. The mentioned Ib protein binds to a cell surface receptor and associates with Ia. The complex is endocytosed. The membrane channel created by Ib enables Ia to enter the cytosol and consequently causes depolymerisation of the actin cytoskeleton via ADP-ribosylation [37].
- CPE (enterotoxin) is also a pore-forming toxin able to bind to claudin receptors on the cell surface [38]. It is able to form a hexamer complex, playing an important role in calcium influx. The mentioned influx is dose-dependent and is one cause of activation of calpain and, consequently, cell death. The activity of CPE is the main cause of food-poisoning and non-foodborne diarrhoea [36].
- NetB (necrotic enteritis B-like toxin) is identified in avian necrotic enteritis, causing pore formation. NetB has been shown to have a 38% sequence similarity with CPB [39].
- Perfringolysin O (PFO) is also a pore-forming toxin and has synergistic effects with CPA, and it is considered to be involved in the pathogenesis of gas gangrene [40]. It is able to target red blood cells and causes coagulative necrosis [9].

**Table 2:** Histotoxic activity of *C. perfringens* major toxins [36,41,42,43,44].

Toxin	Location	Biological Activities	Actions
CPA	Chromosomal	Necrotising, haemolytic, contraction of smooth muscle	Phospholipase C, activation of host cell signalling
CPB	Plasmid	Dermonecrosis, enterotoxic, oedema	Formation of pores
ETX	Plasmid	Dermonecrosis, oedema, contraction of smooth muscle	Formation of pores
ITX	Plasmid	Necrotising	ADP-ribosylating activities
PFO	Chromosomal	Necrotising	Formation of pores
CPE	Chromosomal/plasmid	Enterotoxic, erythema	Formation of pores
NetB	Plasmid	haemolytic	Formation of pores

## 3. *Clostridium perfringens*—Epidemiology

*C. perfringens* is associated with myonecrosis and gas gangrene (a highly lethal, necrotising infection of skeletal muscle and subcutaneous tissue that is most commonly caused by *C. perfringens* type A) [5,45,46]. Nowadays, the incidence of gas gangrene remains very low; however, the lethality is still very high [47], even in the case of special treatment, such as antibiotic therapy or hyperbaric oxygen therapy. The gas gangrene lethality reaches 100% if it goes untreated [48,49]. In addition, myonecrosis was considered one of the commonly occurring diseases, especially during wars, until the 1970s, when improved treatment options were introduced. In the case of gas gangrene and myonecrosis, the main aetiological agent is *Clostridium perfringens* type A. However, other clostridia can also be involved in the disease course. The symptoms of gas gangrene are caused by the toxicoinfection of traumatic wounds from spores or vegetative cells. The multiplication of *C. perfringens* cells causes severe necrosis of affected tissues. Clinical symptoms include fever, pain, oedema, and progressive myonecrosis, leading to sepsis, toxic shock, and, finally, death [50]. Sometimes, the mentioned toxinotype A is considered a causative agent of gastroenteric syndromes. However, ascribing an instance of disease to this type is difficult because it is ubiquitous in the environment. Hence, the isolation of *C. perfringens* type A from the gastrointestinal samples may not be associated with the disease course [51]. Hypothetically, the recently described NetF toxin can be an exception here, as it has been associated with canine haemorrhagic gastroenteritis and necrotising enterocolitis in foals [46,52].

Usually, the infection caused by *C. perfringens* toxinotype B is associated with dysentery in sheep (very rarely in cattle and horses). This disease causes intestinal lesions and enterotoxemia when toxins transfer and absorb into the circulatory system. Dysentery in sheep is characterised by necrohemorrhagic enteritis and rarely by focal symmetrical necrosis associated with the action of the CPB and ETX toxins [53,54,55]. Some of the literature speculates that *C. perfringens* toxinotype type B may be associated with the pathogenesis of multiple sclerosis (MS) because antibodies of ETX serum have been noticed in patients with MS [56]. Ma et al. [57] recently described their findings on a possible association of MS with ETX-producing *C. perfringens* strains, which are biologically plausible pathogens in the mentioned disease, as they trigger inflammatory demyelination in the context of circulating myelin autoreactive lymphocytes.

*C. perfringens* toxinotype C causes necrotising enteritis and enterotoxaemia in many mammalian species of animals and in humans, especially neonates [58,59]. It is hypothesised that the main cause of neonate affection is related to CPB’s sensitivity to trypsin, which provides a natural defence against the disease. The colostrum consumed by neonates is considered a potential trypsin inhibitor, making them more susceptible to the activity of CPB [46]. Rare Type C infection cases are described in people suffering from diabetes and other pancreatic conditions. Type C disease has an acute or per-acute course and is highly fatal [60,61]. Symptoms such as diarrhoea and abdominal pain are usually observed, and neurological damage and sudden death can also be sometimes seen in animals. Pathologically, the disease is characterised by necrotising lesions, and microscopic lesions are characterised by damage caused by severe necrotising enteritis or enterocolitis [62]. Other nonspecific lesions can also be observed, e.g., lesions outside the digestive tract manifesting with circulatory disturbances, including serosal congestion and haemorrhage, oedema, and pulmonary congestion. In the 1960s, a high prevalence of enteritis necroticans, known as PigBel disease, was also observed, caused by toxinotype C with a high prevalence in Papua New Guinea [63]. PigBel occurred mostly in malnourished children with low levels of trypsin caused by poor diet and the consumption of large amounts of sweet potatoes, consisting of high amounts of trypsin inhibitors [62,63,64].

*C. perfringens* toxinotype D is associated with enterotoxemia and enterocolitis symptoms in sheep, goats, and, rarely, cattle. Enterotoxemia is observed in cattle and sheep, manifesting with lesions in the brain and other extra-intestinal organs, with cases of intestinal lesions only rarely observed. The infection of goats with this toxinotype causes enterocolitis with or without enterotoxemia. The main predisposing factor for the disease is considered the sudden ingestion of feeds rich in highly fermentable carbohydrates [46,54].

The epidemic role of *C. perfringens* type E strains in humans is not fully explained. Only a few cases of disease symptoms associated with these strains have been described in animals [46,65].

*C. perfringens* type E is mainly isolated from the intestinal content of sick animals. Because this toxinotype is a normal inhabitant of the intestinal microflora of healthy individuals and many animal species, the occurrence of these strains is not unequivocal with disease symptoms [46].

Strains belonging to *C. perfringens* type F are able to produce the α-toxin and CPE upon sporulation. Previously, these strains were classified as toxinotype A. These strains are known to be responsible for human food-poisoning and non-foodborne diarrhoea but also antibiotic-associated diarrhoea [5].

The epidemiological evidence linking these strains to *C. perfringens* food-poisoning and some cases of non-food related diarrhoea is very clear and well-described [5,66]. Concentrated culture supernatants from two wild-type sporulating strains of what is now referred to as *C. perfringens* type F have been shown to cause fluid accumulation and mucosal damage in a rabbit intestinal loop disease model, in contrast to isogenic *cpe* mutants isolated by allelic exchange into these various strains of *C. perfringens*. Supplementing the mutants with the wild-type *cpe* gene restored the ability to cause fluid accumulation and mucosal damage. This provided clear evidence that CPE is essential for disease in a suitable animal model. *Clostridium perfringens* toxinotype F is considered the second-most common foodborne pathogenic agent in the USA. It is estimated that about one million people suffer from foodborne illness caused by this pathogen each year. It causes losses of approx. $400 million yearly [5,67]. Toxinotype F is able to produce highly resistant spores, which promotes their persistence in improperly stored or undercooked foods, very often in large pieces of meat, which are frequently associated with outbreaks. The doubling time of *C. perfringens* cells is very short in comparison to the other clostridia. In incompletely cooked food, where the vegetative microflora is inactivated (especially under pasteurisation temperatures), spores are able to germinate very quickly and lead to a sufficient bacterial burden to trigger foodborne illness. *C. perfringens* type F food poisoning is initiated by the consumption of food contaminated with a large number of vegetative cells [67]. After rapid multiplication of these vegetative cells, toxinotype F sporulates and can produce CPE in the intestines. In the mentioned in vivo sporulation, lysing cells sporulate and release CPE into the intestinal lumen. The symptoms of this food poisoning typically include diarrhoea and abdominal cramps. Usually, the symptoms develop within 12–24 h and resolve without complications within a day. However, a fatal disease course can be observed in older adults or weakened people. Moreover, some *C. perfringens* type F outbreaks have been observed in psychiatric facilities as well as the fatal course of the illness in relatively younger and physically strong people [46]. These fatalities usually occur in people with pre-existing constipation or faecal impaction as a side effect of psychoactive medicines. In the mentioned cases, CPE-induced diarrhoea, which normally removes this toxin from the intestines, was not observed. Therefore, the CPE would be much more likely to be absorbed into the circulatory system and then consequently bind to organs such as the liver and kidneys, causing fatal enterotoxemia. It is estimated that toxinotype F causes about 5–15% of non-foodborne GI disease cases, including antibiotic-associated diarrhoea (AAD) as well as sporadic diarrhoea. Usually, the mentioned CPE-associated AAD cases have a long duration—even up to several weeks—with a much more severe course than in the case of food poisoning. AAD cases are most often observed in hospital environments. It is interesting that type F food poisoning isolates carry the CPE genes on their chromosomes, while non-foodborne isolates carry the CPE genes on plasmids, and their spores are considered more sensitive [68].

*C. perfringens* type G strains are indicated as aetiological agents of avian necrotic enteritis (NE). Avian NE is considered one of the most prevalent poultry diseases, causing very high economic losses. The main predisposing factor for NE is recognised infection by *Eimeria* spp. NE is most frequently observed in chickens. However, many cases have also been reported in other avian species, such as geese, ostriches, turkeys, quail, bluebirds, lorikeets, crows, etc. Usually, NE is reported in 2–6-week-old broiler chickens. This period of early life, before the immune system matures, is predisposed to the occurrence of the illness [46,69]. However, some cases are occasionally observed in older chickens [70]. NE can have a sub-clinical course, where weight gain is seriously affected, or a course with clinical signs. The most characteristic symptoms of NE are the reluctance to move, decreased appetite, diarrhoea, and dehydration. Pathomorphological changes are mainly observed in the jejunum and ileum, while the duodenum and ceca can also be affected, and gas distention can be observed in the intestines. The intestines are usually full of dark-brown semi-liquid content, and blood is rarely observed [70]. Subacute and chronic cases of NE are characterised by similar lesions. However, the intestinal wall is usually thickened. In the subclinical form of NE, multifocal mucosal ulcerations are characteristic. In some chickens with NE, cholangiohepatitis can also occur in the livers and manifest with firm, pale, and enlarged livers with numerous, diffuse yellow foci of necrosis [46,70].

A very significant problem is the growing antibiotic resistance of *C. perfringens* strains. Not only is antimicrobial use associated with increased antimicrobial resistance among bacterial pathogens, but selection due to antimicrobial use may also contribute to a positive or negative correlation with virulence determinants [71]. Resistance and virulence may not always be independent properties, and their relationship may play an important role in the pathogenesis of *C. perfringens* infection [72].

Complex studies on antibiotic resistance have been published or investigated in several countries like: Belgium, Taiwan, Egypt, Korea, Brazil, and Canada. All of these studies investigated chicken and turkey isolates [73]. Most of the described results reported the resistance to antimicrobial agents frequently used as growth promoters (bacitracin, avilamycin, virginiamycin) in addition to coccidiostats (salinomycin, monensin), which are also active against *Clostridium* spp. in the intestines [74]. A Taiwanese study also reported that MIC50 values of erythromycin and lincomycin for *C. perfringens* isolated from intestinal samples with severe lesions were significantly higher compared to those with mild lesions [75]. On the other hand, a Korean report on resistance patterns between isolates from healthy and sick flocks found no difference [73]. Reports from Canada showed that *C. perfringens* isolates from chickens and turkeys seemed more resistant against bacitracin and virginiamycin in comparison to bovine and porcine isolates [76] but not for other antimicrobials tested. Scandinavian and Belgian studies have identified the *tetP* (B), *tet* (M), *tetA* (P), and *tetB* (P) genes among tetracycline-resistant isolates. In addition, a Belgian report showed strains with the *lnu* (A) and *lnu* (B) genes to be associated with low-level resistance against lincomycin [73].

## 4. *Clostridium perfringens*—Molecular Diversity

*Clostridium perfringens* is known as an important cause of diseases in humans and animals. However, reports about genetic diversity and characterisation are currently limited.

The association between intestinal disorders and *C. perfringens* is clearly defined. However, the underlying factors influencing the pathology are not completely understood. Knowing the genes that participate in the sporulation, germination, toxin production, oxygen tolerance, antimicrobial resistance, and other novel virulence factors may lead to better prevention of *C. perfringens*–associated intestinal diseases, whether it be in humans or animals.

Kiu and Hall [9] characterised the genomic variation, pangenomic diversity, and key virulence traits of 56 *C. perfringens* strains that included 51 public and 5 newly sequenced genomes using whole genome sequencing (WGS). Their study found that *C. perfringens* has a pangenome of 11,667 genes, with 12.6% being core genes, which was identified as the most divergent pangenome of a single Gram-positive species reported up to now. The performed computational analyses determined the phylogeny of *C. perfringens* (16S rRNA gene) in relation to some species of *Clostridium*. *Clostridium baratii* and *C. sardiniense* are considered the closest relatives. Virulence factor profiling confirmed the presence of well-characterised exotoxin genes associated with *C. perfringens*, including α-toxin (*cpa* or *plc*), enterotoxin *(cpe),* and perfringolysin O (*pfo* or *pfoA*). However, interestingly, the researchers did not find a close correlation between the encoded toxin type and disease phenotype. Moreover, the genomic analysis provided by Kiu and Hall [9] indicated significant horizontal gene transfer events, as defined by the presence of prophage genomes, and, notably, the absence of CRISPR defence systems in >70% (40/56) of the strains. The authors discovered that mechanisms of antimicrobial resistance, tetracycline resistance genes (*tet*), and anti-defensins genes (*mprF*) were consistently detected (*tet*: 75%; *mprF*: 100%). However, pre-antibiotic era strain genomes did not encode for *tet*, suggesting antimicrobial selection pressure in the evolutionary history of *C. perfringens* over the last 80 years [9].

The virulence of a *C. perfringens* strain is often dependent on the toxins encoded by large plasmids that range in size from ~45 to ~140 kb. These plasmid-encoded toxins are often closely associated with mobile elements. A *C. perfringens* strain can carry up to three different toxin plasmids, with a single plasmid carrying up to three distinct toxin genes [41].

*Clostridium perfringens* isolates can carry up to 10 plasmids, which significantly influence the pathogenic properties. A study by Gulliver et al. [77] suggested that toxin genes that are known to be responsible for the course of diseases are located on plasmids. The authors characterised these plasmids with a novel apparatus that may contribute to the dissemination of genes within the species and between *C. perfringens* and other species (specifically *C. botulinum*). The extensively analysed plasmids, in particular isolates, suggested key relationships between host species and *C. perfringens* strains and identified a putative novel conjugation locus (Bcp) with sequence similarity to *C. botulinum* plasmids. The authors identified the first putative non-conjugative enterotoxin (CPE)-encoding plasmids. They conducted a whole genome analysis of 464 strains and extracted 1045 contigs encoding a plasmid replication (*rep*) gene or plasmid-encoded toxin genes. The authors observed that plasmid and chromosomally encoded toxin genes correlate with the host or source of the isolate and are necessary for the disease type.

Abdel-Glil et al. [78] conducted an analysis of 206 publicly available genomes of *C. perfringens* strains from various ecological niches. Their analysis unravelled five stable phylogroups of *C. perfringens*. According to the authors, phylogroup I is mainly involved in foodborne diseases in humans and shows unique genomic features, such as the strong presence of insertion sequences and excessive loss of genes involved in metabolism and virulence. Similar characteristics have been reported in other bacteria that have evolved to specialise towards a particular habitat, e.g., *Streptococcus equi* and *Shigella* species [78,79]. The loss of genes in this phylogroup contrasts with the majority of phylogroup II strains (26 out of 32) that have been isolated from intestinal lesions in horses and dogs, which appear to be geared towards acquiring new genetic material. The data presented by Abdel-Glil et al. [78] showed that even for sporulating species, such as *C. perfringens*, the occupation of certain habitats can have a strong impact on phylogeny.

## 5. Conclusions

The short generation time, the multi-production capability of toxins and heat-resistant spores, the location of many virulence genes on mobile genetic elements, and the inhabitance of this opportunistic pathogen in different ecological niches make *C. perfringens* a very important microorganism from the point of view of public health protection. The epidemiological evidence for the association of these strains with *C. perfringens*–meditated food poisoning and some cases of non-foodborne diseases is very clear and well-documented. However, the genetic diversity and physiology of *C. perfringens* should continue to be studied in order to confirm the importance of suspected novel virulence traits. A very significant problem is the growing antibiotic resistance of *C. perfringens* strains. Not only is the use of antimicrobials associated with increased antimicrobial resistance among bacterial pathogens, but selection for antimicrobial use may also contribute to a positive or negative correlation with determinants of virulence. It is important the establish future complex platforms for *C. perfringens* diseases prevention (e.g., microbiota-based therapeutics, vaccines, phage therapy). Furthermore, understanding the genomes (HGT, the role of phages) via novel molecular biology tools will probably enable easier epidemiological investigation.

## Figures and Tables

**Table 1:** Toxinotyping scheme of *C. perfringens* according to Rood et al. [5,11,12].

Toxinotype	α-Toxin CPE	β-Toxins CPB	ε-Toxins ETX	ι-Toxins ITX	CPE Enterotoxin	NetB Necrotic Enteritis B-like Toxin	Diseases in Humans and Animals
Genes	*cpa/plc*	*cpb*	*etx*	*iap/ibp/itx*	*cpe*	*netB*	*-*
A	+	−	−	−	−	−	Gas gangrene in humans and animals; necrotic enteritis in fowls and piglets; human food poisoning and antibiotic-associated diarrhoea
B	+	+	+	−	−	−	Haemorrhagic enteritis in calves, foals and sheep; dysentery in lambs
C	+	+	−	−	±	−	Enterotoxemia in sheep; necrotising enteritis in humans (pigbel), pigs, calves, goats, and foals
D	+	−	+	−	±	−	Enterotoxemia in lambs (pulpy kidney disease), goats, and cattle
E	+	−	−	+	±	−	Enterotoxemia in calves and lambs
F	+	−	−	−	+	−	Food and feed poisoning of humans and animals
G	+	−	−	−	−	+	Avian necrotic enteritis

## Data Availability

Not applicable.
